# Excess risk of heat-related hospitalization associated with temperature and PM_2.5_ among older adults

**DOI:** 10.1097/EE9.0000000000000451

**Published:** 2025-12-30

**Authors:** Lauren Mock, Rachel C. Nethery, Poonam Gandhi, Ashwaghosha Parthasarathi, Melanie Rua, David Robinson, Soko Setoguchi, Kevin Josey

**Affiliations:** a Department of Biostatistics, Harvard T.H. Chan School of Public Health, Boston, Massachusetts; b Rutgers University Institute for Health, Healthcare Policy, and Aging Research, New Brunswick, New Jersey; c Department of Geography, Rutgers University, Piscataway, New Jersey; d Department of Medicine, Rutgers Robert Wood Johnson Medical School, New Brunswick, New Jersey; e Department of Biostatistics and Informatics, Colorado School of Public Health, Aurora, Colorado

**Keywords:** Air pollution, Case-crossover, Exposure synergism, Heat-related illness, Medicare, Temperature

## Abstract

**Background:**

With rising temperatures and an aging population, understanding how to prevent heat-related illness among older adults will be increasingly crucial. Despite biological plausibility, no study to date has investigated how exposure to fine particulate matter air pollution (PM_2.5_) may contribute to the risk of hospitalization with a diagnosis code indicating heat-related illness, referred to as heat-related hospitalization. This study aims to fill this gap by investigating the independent and combined effects of temperature and PM_2.5_ exposures on heat-related hospitalization risk.

**Methods:**

We identified Medicare fee-for-service beneficiaries in the contiguous United States who experienced a heat-related hospitalization between 2008 and 2016. Using a case-crossover design and fitting Bayesian conditional logistic regression models, we characterized the associations of temperature and PM_2.5_ exposures with heat-related hospitalization. We then estimated the relative excess risk due to interaction to quantify the additive-scale interaction of simultaneous exposure to heat and PM_2.5_.

**Results:**

We observed 112,969 heat-related hospitalizations among 29,345,820 Medicare beneficiaries in the study sample. Fixing PM_2.5_ at the case day median, the odds ratio for increasing temperature from its case day median to the 95th percentile was 1.05 (95% CI: 1.03, 1.06). Fixing temperature at the case day median, the odds ratio for increasing PM_2.5_ from its median to the 95th percentile was 1.01 (95% CI: 0.99, 1.04). The risk due to interaction for simultaneous median-to-95th percentile increases in temperature and PM_2.5_ was 0.03 (95% CI: 0.01, 0.06).

**Conclusions:**

Our study is the first to observe synergism between temperature and PM_2.5_ exposures associated with the risk of heat-related hospitalization. These findings highlight the importance of considering air pollution in effective public health and clinical interventions to prevent heat-related illness.

**What this study adds**

While previous studies have identified synergism between temperature and fine particulate matter (PM_2.5_) on mortality, no study to date has investigated the role of PM_2.5_ in hospitalizations for heat-related illness, referred to as heat-related hospitalizations. Among older Americans, we found no independent effect of PM_2.5_ but identified an interaction between simultaneous acute temperature and PM_2.5_ exposures, suggesting that PM_2.5_ exacerbates heat-related hospitalization risk. Our findings emphasize the importance of air pollution considerations in heat preparedness.

## Introduction

Heat is a leading cause of weather-related illness and death, and its impact is expected to intensify with climate change.^1^ Previous research suggests that each year, thousands of deaths in the United States are directly or indirectly attributable to heat exposure.^2^ Extreme heat events have been associated with increased hospitalizations for conditions that may be triggered or exacerbated by heat, such as respiratory,^3^ renal,^4^ and cardiovascular^5^ diseases, as well as conditions that occur as a direct result of heat exposure,^6^ which are broadly referred to as heat-related illness. Heat-related illness ranges in severity from mild conditions such as heat cramps and syncope to severe, life-threatening conditions like heat stroke. Many of these outcomes are preventable through proactive measures such as avoiding direct sun, maintaining hydration, or temporarily relocating to public cooling centers—actions often prompted by heat alerts.^7,8^ However, despite these preventive efforts, heat-related emergency department visits in the United States reached record levels during the summer of 2023.^9^

Older adults are particularly vulnerable to heat due to impaired thermoregulation, social isolation, a higher prevalence of comorbidities, and the use of medications that increase heat sensitivity.^6,8,10^ As the US population ages and extreme heat events become more frequent and intense,^11^ it will become increasingly critical to understand and implement strategies to protect older Americans from heat-related illness.

Previous studies have identified synergistic effects between acute exposure to heat and particulate matter (PM) air pollution on mortality^12–14^ and on certain cause-specific hospitalizations.^15^ These investigations of synergism are especially critical since daily temperature and PM_2.5_ levels are often correlated across much of the United States, even after accounting for seasonal and long-term trends, leading to frequent simultaneous high exposures.^16^ Despite biological plausibility, no study has yet investigated the role of PM in heat-related hospitalizations (HRH). PM_2.5_ is small enough to penetrate the lungs and bloodstream, affecting several organ systems. Evidence from epidemiological and toxicological studies shows that acute exposure to PM_2.5_ can impair vascular function, trigger oxidative stress and inflammation, and increase blood pressure.^17,18^ These physiological responses may hinder the body’s ability to thermoregulate in hot conditions, potentially increasing the risk of heat-related illness when PM_2.5_ levels are high. Understanding the impact of PM_2.5_ on heat-related illness is critical for improving heat alert systems and guiding preparedness efforts.

To address this gap, we investigate the independent and combined effects of temperature and PM_2.5_ on HRHs, defined as hospitalizations due to heat-related illness, among older Medicare fee-for-service (FFS) beneficiaries. Specifically, our analysis focuses on inpatient hospitalizations for heat-related illness during the warm season (June–September) from 2008 to 2016. We employ a case-crossover design and a Bayesian analytic approach to: (1) reduce bias from unmeasured, time-invariant confounders,^19,20^ and (2) provide interpretable inferences using posterior distributions generated by Markov chain Monte Carlo sampling. We model nonlinear associations between the exposures and outcomes, and we assess synergy between heat and PM_2.5_ by estimating the relative excess risk due to interaction (RERI), an additive-scale interaction quantity.

## Methods

### Study population and case definition

We assessed records from a 50% random sample of Medicare FFS beneficiaries aged 65 and older who resided in the contiguous United States and were continuously enrolled in Parts A, B, and D for at least 12 months between 2008 and 2016. These records include data on beneficiaries’ sex, age, race/ethnicity, Medicaid eligibility status, ZIP code of residence, inpatient admission and discharge dates, and date of death, if applicable. We identified inpatient hospitalizations with a primary or secondary diagnosis of heat-related illness, based on International Classification of Diseases (9th and 10th revisions) diagnosis codes; detailed International Classification of Diseases-9 and -10 codes are provided in the Table S1 of Supplementary Material; https://links.lww.com/EE/A395. In the United States, the majority of unscheduled inpatient hospitalizations follow an emergency room visit, although some admissions originate with physician referrals from outpatient settings.^21^ We refer to the composite of these indications as HRH. Our analysis is limited to individuals who experienced an HRH during the warm season (June–September), and we included only each beneficiary’s first HRH during the study period, excluding any subsequent events.

### Exposure assessment

Daily maximum temperature data were obtained from the Parameter-elevation Relationships on Independent Slopes Model,^22,23^ which estimates temperature across the contiguous United States using surface station data and geographic factors such as elevation, coastal proximity, and atmospheric conditions.^23^ Temperature estimates are available at a 4-km resolution. For each ZIP code, we assigned the temperature estimate from the Parameter-elevation Relationships on Independent Slopes Model grid cell whose centroid was nearest to the ZIP code’s population-weighted centroid. Daily PM_2.5_ concentrations were derived from a validated ensemble model that integrates ground monitoring data, chemical transport models, satellite observations, and land-use variables.^24,25^ These estimates, available at a 1-km resolution, were aggregated to the ZIP code level by averaging values from grid cells whose centroids fell within each ZIP code.

For each individual and date, exposure was defined as: (1) the daily maximum temperature in the individual’s residential ZIP code on that date, and (2) the three-day average PM_2.5_ concentration in the individual’s residential ZIP code, calculated as the mean concentration from the case day and the two preceding days. We used an extended exposure window for PM_2.5_ to account for the delayed health effects that might be incurred by air pollution exposures, which has been observed previously.^26,27^ As some studies have also identified lagged effects of heat on hospitalizations,^28,29^ we conducted a sensitivity analysis using three-day exposure windows for both temperature and PM_2.5_.

### Statistical analysis

We used a case-crossover design to evaluate the effects of acute heat and PM_2.5_ exposures among individuals who experienced an HRH, with each individual serving as their own control. The case day was defined as the admission date of an individual’s first HRH during the study period. Control days were assigned bidirectionally within the same calendar month and matched to the same day of the week as the case day, with up to four matched control days per case selected before or after the hospitalization date. To eliminate potentially undue influence from extreme PM_2.5_ concentration outliers, we calculated the 95th percentile of three-day PM_2.5_ exposures across all case and control days and trimmed, or excluded, observations exceeding this threshold.^30,31^ We also conducted a sensitivity analysis using the 99th percentile as the threshold for trimming.

We implemented the Bayesian analogue to conditional logistic regression using the brms R package^32^ to estimate the associations of temperature and PM_2.5_ with HRH. The primary model comprised three terms: a natural cubic spline for temperature, a natural cubic spline for PM_2.5_, and a linear interaction term. As a sensitivity analysis, we also applied a tensor product spline approach to enable a more complex, nonlinear interaction between the exposures. Detailed descriptions of both approaches are provided in the Supplementary Material; https://links.lww.com/EE/A395.

We estimated the odds of HRH under various temperature and PM_2.5_ scenarios, reporting posterior means and 95% Bayesian credible intervals (CIs) derived from the posterior distribution samples of the Markov chain Monte Carlo. While the measures of association from conditional logistic regression models are typically evaluated on a multiplicative scale, multiplicative interactions can be challenging to interpret in a public health context.^33^ Instead, we assessed the interaction between heat and PM_2.5_ on an additive scale by estimating the relative excess RERI.^34,35^ The RERI quantifies the increased risk associated with simultaneous exposure to heat and PM_2.5_ beyond the sum of their individual effects. Because HRH is a rare outcome, the odds ratio-based RERI estimate should closely approximate the conventional RERI, which is a function of relative risks.^35^

To estimate the RERI for two continuous exposures, we selected the median and 95th percentile values from the distributions of temperature and three-day average PM_2.5_ concentrations on case days as contrast levels. This approach to assessing synergism between exposures has been applied in similar epidemiological studies.^36,37^ Additional methodological details are provided in the Supplementary Material; https://links.lww.com/EE/A395. Finally, we also report the interactions between temperature and PM_2.5_ on a multiplicative scale as recommended by VanderWeele and Knol^35^ as well as Vandenbroucke et al,^38^ estimating this measure using posterior means and 95% Bayesian CIs from the linear interaction coefficient.

All analyses were completed in R (version 4.4.0; Vienna, Austria)^39^; code to reproduce these analyses is available at https://github.com/laurenmock/temp_pm25_heat-hosp.

Our study aims and data stewardship plan were approved by the Rutgers University Institutional Review Board (Pro20170001685). Medicare patient data are protected by our data usage agreement with the Centers for Medicare and Medicaid, and were stored and analyzed with minimal risk of breach of data confidentiality. Use of Medicare data does not require informed consent from individual beneficiaries.

## Results

### Main analysis

Among 29,345,820 Medicare beneficiaries in our sample, we observed at least one HRH among 112,969 unique individuals. Each individual’s first HRH served as a case in our case-crossover study. Heat-related illness was the primary diagnosis for 60,053 hospitalizations (53.2%) and the secondary diagnosis for the remaining hospitalizations. The mean length of stay in the hospital was 3.5 (standard deviation: 4.5) days. The characteristics of the individuals who experienced an HRH are detailed in Table 1. Most of the hospitalized individuals were women (68.2%) and white (82.3%), with 22.9% eligible for Medicaid. The mean age at the time of hospitalization was 81.7 years. On case and control days, the mean maximum daily temperatures were 29.2 °C and 29.1 °C, respectively. Before trimming, the case day distribution of three-day PM_2.5_ concentrations was heavily right-skewed, with exposures as high as 120.4 µg/m^3^. Case and control days with three-day PM_2.5_ exposures above 18.4 µg/m^3^ (the 95th percentile of the distribution) were trimmed; this resulted in a mean PM_2.5_ concentration of 9.4 µg/m^3^ on case days and 9.3 µg/m^3^ on control days. The full distributions of temperature and PM_2.5_ case day exposures are displayed in Figures S1 and S2 in the Supplementary Material; https://links.lww.com/EE/A395.

**Table 1.** Characteristics of individuals included in the analysis and exposures on case and control days

	N (%)
Total	112,969
Sex	
Male	35,930 (31.8%)
Female	77,039 (68.2%)
Age category	
65–74	19,165 (17%)
75–84	45,868 (40.6%)
85+	47,936 (42.4%)
Race/ethnicity	
White	92,975 (82.3%)
Black/African-American	13,123 (11.6%)
Hispanic	3,178 (2.8%)
Asian/Pacific Islander	2,006 (1.8%)
American Indian/Alaska Native	650 (0.6%)
Other	1,037 (0.9%)
Medicaid eligibility	
Ineligible	87,059 (77.1%)
Eligible	25,910 (22.9%)
	**Mean (SD**)
Daily maximum temperature (^°^C)	
Case days	29.2 (5.0)
Control days	29.1 (5.1)
Three-day PM_2.5_ (µg/m^3^)	
Case days	9.4 (3.7)
Control days	9.3 (3.6)

Case day temperature exposure is defined as the maximum daily temperature on the day of hospitalization. Case day PM_2.5_ exposure is defined as the three-day mean PM_2.5_ exposure on the day of hospitalization and the two preceding days.

SD, standard deviation.

**Figure 1.**
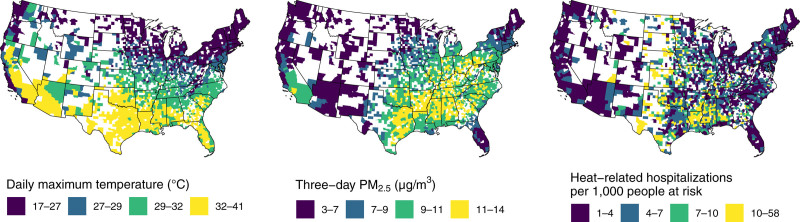
From left to right, aggregated county-level maps of (1) mean case day maximum temperature, (2) mean case day three-day PM_2.5_ concentrations, and (3) number of heat-related hospitalizations per 1000 Medicare FFS beneficiaries at risk of hospitalization during the study period. Counties with 10 or fewer heat-related hospitalizations observed during the study period are suppressed for confidentiality and are displayed in white.

Temperature exposure on case days was highest in the South and Southwest regions of the United States (Figure 1, left panel), while PM_2.5_ exposure concentrations were highest in the South, Midwest, and Southern California (Figure 1, middle panel). The rates of HRH per 1000 Medicare FFS beneficiaries at risk varied substantially across the United States, with the highest rates in the Central United States (Figure 1, right panel).

**Figure 2.**
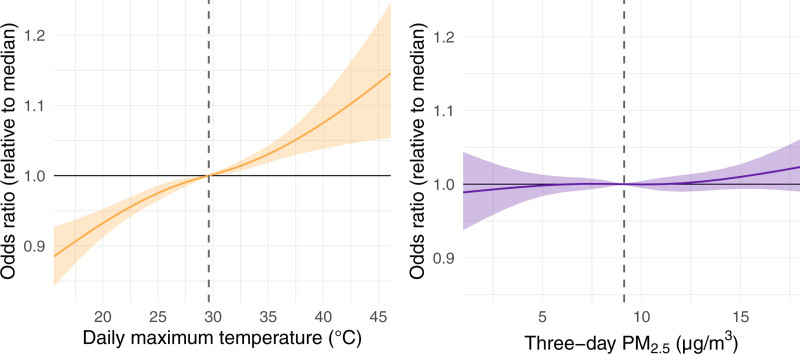
Independent nonlinear effects of temperature and PM_2.5_ on heat-related hospitalization. The left panel displays the odds ratio of heat-related hospitalization comparing the median case day temperature (29.6 °C), shown with a vertical dashed line, to various alternative temperatures across the *x* axis, while holding PM_2.5_ exposure fixed at the median (8.9 µg/m^3^). The right panel displays the odds ratio of heat-related hospitalization comparing the median case day PM_2.5_ exposure, shown with a vertical dashed line, to various alternative concentration levels across the *x* axis, while holding temperature exposure fixed at the median.

**Figure 3.**
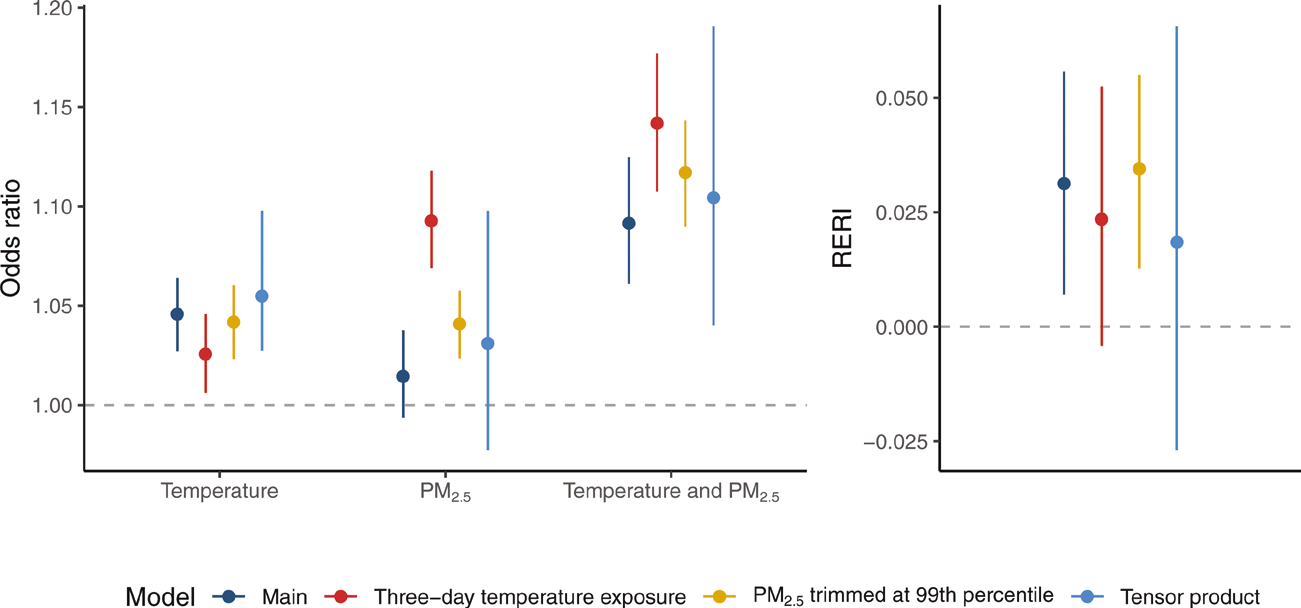
Comparison of the main and sensitivity analyses. The left panel displays odds ratios with 95% Bayesian credible intervals associated with (1) increasing temperature from the median to the 95th percentile while holding PM_2.5_ at the median, (2) increasing PM_2.5_ from the median to the 95th percentile while holding temperature at the median, and (3) increasing both temperature and PM_2.5_ from the median to the 95th percentile simultaneously. The right panel displays the relative excess risk due to interaction (RERI) with 95% Bayesian credible intervals. The same exposure contrasts were used across models. All numerical results are presented in Table S2 in the Supplementary Material; https://links.lww.com/EE/A395.

Figure 2 shows the independent effects of heat and PM_2.5_ on HRH, with PM_2.5_ held at its case day median to examine the effect of heat, and temperature held at its case day median to examine the effect of PM_2.5_. Maximum daily temperature exhibited a strong positive association with odds of HRH (Figure 2, left panel). Specifically, when PM_2.5_ concentrations are fixed at 8.9 µg/m^3^, increasing the temperature from 29.6 °C (median) to 36.9 °C (95th percentile) yields an odds ratio of 1.05 (95% CI: 1.03, 1.06) (Figure 3 and Table S2; https://links.lww.com/EE/A395). Although a positive association was observed between three-day PM_2.5_ and HRH, credible intervals were wide and covered one across the range of PM_2.5_ levels (Figure 2, right panel). When temperature was fixed at 29.6 °C, an increase in PM_2.5_ concentrations from 8.9 µg/m^3^ (median) to 16.1 µg/m^3^ (95th percentile) yielded an odds ratio of 1.01 (95% CI: 0.99, 1.04) (Figure 3 and Table S2; https://links.lww.com/EE/A395).

**Figure 4.**
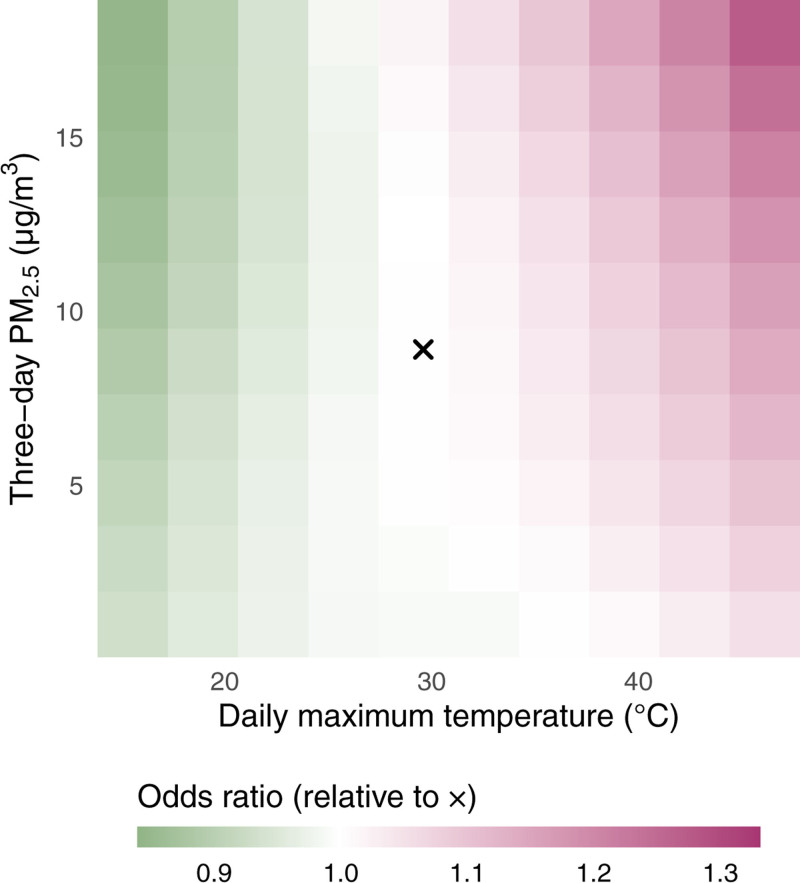
Synergistic effects of temperature and PM_2.5_ across a range of exposure values. The tile color indicates the odds ratio of heat-related hospitalization for a given pair of temperature and PM_2.5_ exposures versus the median temperature and PM_2.5_ exposures (marked on the grid with an ×).

Furthermore, we observed an additive interaction between temperature and PM_2.5_. A simultaneous increase in both temperature and PM_2.5_ from their median values to their respective 95th percentile quantities yielded an odds ratio of 1.09 (95% CI: 1.06, 1.12) (Figure 3 and Table S2; https://links.lww.com/EE/A395). Using these odds ratios to approximate the RERI, we found that the approximate risk increase was 0.03 (95% CI: 0.01, 0.06) (Figure 3 and Table S2; https://links.lww.com/EE/A395), indicating synergism between the two exposures resulting in excess HRHs. We visualized synergism between these two exposures by comparing the odds of HRH at median exposure levels to the odds of HRH across a grid of exposure values (Figure 4). Temperature was strongly associated with HRH, but on hot days, the odds of HRH also increased with higher concentrations of PM_2.5_. On cooler days, however, we see a small protective effect of PM_2.5_. Since temperature and PM_2.5_ were often correlated (as shown in Figure S2; https://links.lww.com/EE/A395), we have limited data to assess the effect of elevated PM_2.5_ at lower temperatures during the warm season.

Although we were primarily interested in assessing the additive interaction with the RERI, we also estimated the interaction on a multiplicative scale. The estimated odds ratio for the linear interaction term was 1.0003 (95% CI: 1.0001, 1.0006). Both the additive and multiplicative interaction estimates were positive, with credible intervals that did not cover the null value.

### Sensitivity analyses

To investigate the interaction between temperature and PM_2.5_ further and assess the robustness of our findings, we fit three sensitivity analyses. Note that we used the median and 95th percentile contrasts defined in the main analysis across all sensitivity analyses to allow for direct comparisons across models.

First, we extended the temperature window from one day to three days and observed attenuated estimated associations between temperature and HRH with wider credible intervals relative to the main analysis (Figure 3 and Figures S3 and S4; https://links.lww.com/EE/A395). Second, we modified the trimming threshold from the 95th percentile of the three-day PM_2.5_ distribution (18.4 µg/m^3^) to the 99th percentile (23.7 µg/m^3^). The findings indicate a much stronger positive association between PM_2.5_ and HRH than that seen in the main analysis, with narrow credible intervals that do not include one (Figure 3 and Figure S3; https://links.lww.com/EE/A395). Finally, we developed an additional model using a tensor product to allow for a more flexible, nonlinear interaction between these exposures. This approach resulted in more linear estimates of the independent effects of temperature and PM_2.5_ compared with the other analyses (Figure S3; https://links.lww.com/EE/A395). The estimated RERIs from all three analyses were positive, but the credible intervals from the sensitivity analyses with a longer temperature exposure and a tensor product included the null value of zero (Figure 3).

## Discussion

### Summary and key findings

To our knowledge, this is the first study to investigate the combined (and synergistic) effects of acute exposure to high temperatures and elevated PM_2.5_ on HRHs. Comparing median and 95th percentile exposure levels, we found a positive association between temperature and HRH. The association between PM_2.5_ and HRH was also positive, although the credible interval covered the odds ratio scale null value of one. Finally, we identified evidence of an additive interaction between temperature and PM_2.5_.

Three sensitivity analyses demonstrate robustness in our findings and provide additional insights. Most notably, the analysis with a longer temperature exposure window indicated a weaker relationship between temperature and HRH, suggesting that the effects of temperature on HRH are more immediate. Additionally, the wider credible intervals from the tensor model reflect increased uncertainty in the estimates when allowing for greater flexibility in the interaction structure.

Our findings offer the first empirical, epidemiological evidence that PM_2.5_ may increase the risk of HRH. In contrast to prior work, which has largely framed temperature as a modifier of the health effects of air pollution,^13,40,41^ our focus on explicit HRHs reveals that PM_2.5_ may also modify the health effects of heat.

### Impact

Heat is the deadliest form of extreme weather in the United States,^42^ and rates of heat-related illness continue to climb,^9,43^ even though these illnesses are often preventable.^44,45^ Understanding how to protect vulnerable individuals, including older adults, from extreme heat events is a crucial component of climate change adaptation and an urgent public health priority.^9^

The National Weather Service issues localized heat alerts to initiate individual- and community-level preparations on unusually warm days.^46^ Our findings indicate that additional caution is necessary on hot days when PM_2.5_ levels are high, and it may be useful to consider PM_2.5_ when issuing heat alerts. However, the efficacy of heat alerts alone in minimizing illness and death is uncertain.^47,48^ Previous research highlights that high-risk individuals must recognize their own vulnerabilities to heat, possess the knowledge and resources necessary to protect themselves, and take action to stay safe.^48^ Interestingly, previous studies report that many older adults recognize age as a risk factor for heat-related illness but do not recognize high temperatures as a threat to their personal health.^49^ Clinicians could play an important role in helping these individuals understand their risk.^44^

Our results also emphasize the importance of minimizing personal exposure to particulate matter, particularly on unusually warm days. For example, open windows may allow more ambient particulate matter into homes^50^ and further increase the risk of heat-related illness. These findings also contribute to the growing body of evidence that PM_2.5_ is linked to a range of negative health effects, underscoring the importance of reducing ambient PM_2.5_, particularly with a warming planet.

### Epidemiological evidence and biological plausibility of synergism

This study supports mounting epidemiological evidence of synergism between temperature and PM_2.5_. Several studies have assessed effects on mortality; for example, a case-crossover study from Italy^12^ identified positive but generally nonstatistically significant interactions between temperature and PM_10_, which includes both PM_2.5_ and larger particles, on mortality, and a meta-analysis by Li and colleagues^13^ also reported that extreme temperatures modify the effect of PM_10_ on nonaccidental and cardiovascular mortality. An examination of PM_2.5_ and mortality in US cities^14^ revealed a stronger association between long-term PM_2.5_ exposure and mortality in warmer cities. A smaller number have assessed hospitalization outcomes, including a recent study in New England that identified synergism between short-term exposures to temperature and PM_2.5_ for both respiratory and cardiac hospital admissions.^15^ Our study contributes to this growing body of work and extends scientific understanding of synergism between these short-term exposures to HRHs, providing additional insight into the pathways between simultaneous temperature and air pollution exposures and morbidity and mortality.

Furthermore, it is biologically plausible that PM_2.5_ may interfere with the body’s ability to respond to high temperatures. Simultaneous exposure to heat and PM_2.5_ can exacerbate heat-related adverse health outcomes through shared pathophysiological mechanisms, including autonomic dysfunction and heightened inflammatory responses. Both heat and PM_2.5_ cause autonomic dysfunction by increasing sympathetic drive and suppressing parasympathetic activity.^51,52^ This imbalance leads to a 4%–7% reduction in heart rate variability and an increase in heart rate by 5–9 beats per minute.^53–55^ In addition, blood pressure increases due to autonomic vasoconstriction from PM_2.5_ exposure, with each 10 µg/m^3^ increment correlating to a 9-mm Hg rise in systolic pressure.^56^ Furthermore, increased sweating via sympathetic activation in response to heat elevates blood pressure by increasing plasma viscosity by 2% per °C.^57^ Thus, the combined cardiovascular stress disrupts hemodynamic regulation, compromising the body’s thermoregulatory function and exacerbating heat stress.

Heat and PM_2.5_ can also increase systemic inflammatory responses by forming reactive oxygen species within the cells. The resulting oxidative stress activates redox-sensitive transcription factors, driving the production and release of pro-inflammatory mediators such as tumor necrosis factor-alpha, interleukin-6, and interleukin-1β.^1,58^ A 10-µg/m^3^ increase in PM_2.5_ exposure corresponds to a five-fold rise in these cytokines, and a 2 °C elevation in ambient temperature can boost their expression by up to four-fold.^59,60^ Elevated cytokines also disrupt endothelial homeostasis and increase vascular permeability.^60–62^ The sustained up-regulation of cytokines affects the hypothalamic thermoregulatory center, while impaired vascular permeability decreases the efficacy of the peripheral vasodilatory response and increases the risk of heat-related hospital admissions.^63^

### Limitations and strengths

We acknowledge several limitations to our study. As with any case-crossover study, the results may not fully generalize to other populations or demographic groups; that is, the group of individuals who experienced an HRH is comprised of a small percentage of all Medicare FFS beneficiaries, and results drawn from this sample may not generalize to the overall US population. Furthermore, it is likely that PM_2.5_ partially mediates the relationship between temperature and various health conditions,^64,65^ including heat-related illness, so our estimate of the independent effect of temperature may not fully capture more complex pathways involving PM_2.5_. Consequently, interpreting temperature and PM_2.5_ as entirely independent exposures may be overly simple, as these exposures may influence each other’s effects on health outcomes in complex ways. We attempted to capture this with an interaction term, but this may not sufficiently capture the true relationship between the exposures. Moreover, due to the complexities of quantifying synergism between two continuous, time-varying exposures, our model did not capture daily exposures over time or interactions between lagged exposures that could be incorporated with a distributed lag model.

It is also possible that the observed relationship between temperature and PM_2.5_ is confounded by other pollutants such as ground-level ozone, which may be correlated with PM_2.5_ particularly in warm weather,^66^ although previous studies have produced mixed results.^67^ Finally, maximum daily temperature does not account for other meteorological variables such as humidity and wind speed, which may influence temperature perception; however, previous research indicates the choice of heat metric does not substantially alter epidemiological results.^68^ This study also has several important strengths. We leverage a large and diverse sample of Medicare FFS beneficiaries, enabling robust estimates of risk across a range of environmental conditions. By applying a case-crossover design, the study effectively controls for confounders that are time-invariant or change slowly over time, allowing for more precise inferences of acute exposure effects. Moreover, our use of a flexible statistical model that incorporates both additive and interactive effects of temperature and PM_2.5_ provides valuable insights into the complex, real-world dynamics between these environmental exposures and their impacts on human health. Together, these strengths support the reliability of our findings and their relevance for guiding targeted public health interventions to mitigate the risks of HRH in vulnerable populations.

### Future work

As this study is the first to provide epidemiological evidence for synergism between the effects of temperature and PM_2.5_ on heat-related illness, additional work is needed to understand how these exposures interact. It has been well-established that the dangers of heat vary by geographic region and also time of year,^69^ since individuals adapt behaviorally and biologically to heat. An analysis that accounts for climate type or region may uncover differences in these effects by location. For example, the threat of PM_2.5_ may be greater in areas where fewer people have access to air conditioning and are thus more likely to be exposed to ambient air pollution on hot days. PM_2.5_ is also a diverse category of pollutants that includes a wide array of chemical components from various sources, and these components vary widely by location. Evidence suggests that certain components are more harmful than others.^70–72^ Finally, since this study focuses on older adults, there is a need to assess potential synergism in younger vulnerable individuals, such as children or adults who work outdoors. This study is a first step in understanding the complex synergism between the effects of temperature and PM_2.5_ on heat-related illness, and additional research is essential to understand the risk of simultaneous exposure.

## Conflicts of interest statement

S.S. received research funding from Pfizer Inc., Pfizer Japan, Bristol Myers Squibb, and Daiichi Sankyo and served as a consultant for Pfizer Japan and Merck & Co., Inc. Other authors have no potential conflicts of interest to report.

## ACKNOWLEDGMENTS

All computations presented here were supported by the Office of Research Computing at the Rutgers University Institute for Health, Health Care Policy and Aging Research. Editorial support was provided by Gabriel Shapiro.

## Supplementary Material
