# Supplementary material for: Excess risk of heat-related hospitalization associated with temperature and PM2.5 among older adults
Source: Environ Epidemiol. 2025 Dec 30;10(1):e451. doi: 10.1097/EE9.0000000000000451 (PMC12755698; doi:10.1097/EE9.0000000000000451)
Supplement: Supplementary file 1 [file ee9-10-e451-s001.pdf]

Supplementary materials for "Excess risk of heat-related hospitalization associated with temperature and $PM_{2.5}$ among older adults"

Lauren Mock, Rachel C. Nethery, Poonam Gandhi, Ashwaghosha Parthasarathi, Melanie Rua, David Robinson, Soko Setoguchi, and Kevin Josey

1 Methods

1.1 ICD codes

Heat-related hospitalizations were identified when the primary or secondary diagnosis code of an inpatient hospitalization indicated heat-related illness. We used the following ICD-9 and ICD-10 codes to define heat-related illness:

	ICD-9	ICD-10
Effects of heat and light	992	T67
Exposure to excessive natural heat	E900 (excluding E900.1)	X30
Dehydration	2765	E86

Table S1: Inpatient hospitalization diagnosis codes used to identify heat-related hospitalizations.

1.2 Main analysis: natural cubic splines

We fit natural cubic splines for the effects of temperature and $PM_{2.5}$ using the `ns()` function from the `splines` R package. Each spline had three degrees of freedom, with knot placement performed in an a priori fashion—the default for the `ns()` function.

1.3 Sensitivity analysis: tensor product

As a sensitivity analysis, we also fit a model using a tensor product smooth to more flexibly capture the interaction between temperature and $PM_{2.5}$. Tensor product smooths combine separate marginal smooth terms for each covariate, allowing each to have its own basis functions and smoothness penalties. Specifically, we implemented a `t2`-style tensor product smooth from the `mgcv` R package (Wood et al., 2013), which is

compatible with Bayesian modeling in `brms`. Note that the alternative `te`-style tensor product smooths, which apply penalties differently, are currently not supported in `brms`. For further details and guidance on implementing tensor product smooths (`t2` and `te`) using `mgcv`, see the tutorial by Pedersen et al. (2019).

1.4 OR and RERI estimation details

In a case-crossover study, we use conditional logistic regression to assess how temperature (T) and $PM_{2.5}$ (A) exposures affect the odds of heat-related hospitalization. Let Y_{ij} indicate the outcome for observation $j = 1, 2, \ldots, m_i$ in beneficiary $i = 1, 2, \ldots, n$ (e.g., for each beneficiary, $Y_{ij} = 1$ for the case day and 0 for the control days). We model the log-odds (logit) of the event for a given day's exposures as:

$$\lambda_i(T_{ij}, A_{ij}) = \log\left[\frac{\Pr(Y_{ij} = 1 \mid T_{ij}, A_{ij}, \alpha_i)}{\Pr(Y_{ij} = 0 \mid T_{ij}, A_{ij}, \alpha_i)}\right] = \alpha_i + f(T_{ij}) + g(A_{ij}) + h(T_{ij}, A_{ij}). \quad \text{(S1)}$$

Here $f(\cdot)$ and $g(\cdot)$ are flexible functions (e.g., spline terms) capturing potentially nonlinear effects of temperature and $PM_{2.5}$, and $h(T, A)$ is an interaction term allowing the combined effect of T and A to deviate from additivity on the log-odds scale. In our primary model, we assume a linear interaction $h(T, A) = \gamma T \times A$. In a sensitivity analysis we assume $h(T, A)$ is a tensor product of two spline basis functions. The term α_i is a stratum-specific intercept for each beneficiary, which absorbs baseline risk differences between beneficiaries. Because we condition on each beneficiary, these α_i terms are neither estimated nor reported—in other words, the model does not yield an overall baseline probability of hospitalization. Instead, the regression focuses on estimating the relative effect of exposures within each beneficiary. As a result, the exposure coefficients have a conditional interpretation (i.e., comparing odds of the event between two exposure levels for the same beneficiary) rather than a population-average effect.

The fitted model yields estimates that resemble the log-odds of heat-related hospitalization for any combination of temperature and $PM_{2.5}$ values for a given beneficiary $i = 1, 2, \ldots, n$. Despite the similarities, the fitted values are not actual predictions of the log-odds given the design of the case-crossover study. However, we can still estimate the odds ratio for the outcome from these quantities by contrasting the fitted values of two exposure scenarios. Supposing α_i is the same in both contrasts, exponentiating this difference yields an identifiable odds ratio (OR). For example, consider the distributions of observed case day temperature and $PM_{2.5}$ exposures. Let t_0 be the median temperature and t_1 be a higher temperature (e.g., the 95th percentile), and similarly define a_0 and a_1 for $PM_{2.5}$. The effect of a higher temperature (vs. median temperature) on hospitalization odds, at a common $PM_{2.5}$ level, can be expressed as:

$$OR_{10} = \exp\left[\lambda_i(t_1, a_0) - \lambda_i(t_0, a_0)\right]. \quad \text{(S2)}$$

Without needing to predict α_i, this quantity is identifiable and estimated by

$$\widehat{OR}_{10} = \exp\left[\hat{f}(t_1) + \hat{h}(t_1, a_0) - \hat{f}(t_0) - \hat{h}(t_0, a_0)\right] \quad \text{(S3)}$$

where $\hat{f}(\cdot)$ and $\hat{h}(\cdot)$ are fit with conditional logistic regression. Similarly, we can examine the odds of a heat-related hospitalization attributable to $PM_{2.5}$ by estimating

$$OR_{01} = \exp\left[\lambda_i(t_0, a_1) - \lambda_i(t_0, a_0)\right] \tag{S4}$$

with the plugin estimate

$$\widehat{OR}_{01} = \exp\left[\hat{g}(a_1) + \hat{h}(t_0, a_1) - \hat{g}(a_0) - \hat{h}(t_0, a_0)\right]. \tag{S5}$$

Like $\hat{f}(\cdot)$ and $\hat{h}(\cdot)$, $\hat{g}(\cdot)$ is fit with conditional logistic regression. Finally, we can also examine the joint effect of high temperature and high $PM_{2.5}$ by comparing the odds of an event where both exposures are high to a baseline odds when both exposures are at median levels. This quantity is identified as

$$OR_{11} = \exp\left[\lambda_i(t_1, a_1) - \lambda_i(t_0, a_0)\right] \tag{S6}$$

which is estimated with

$$\widehat{OR}_{11} = \exp\left[\hat{f}(t_1) + \hat{g}(a_1) + \hat{h}(t_1, a_1) - \hat{f}(t_0) - \hat{g}(a_0) - \hat{h}(t_0, a_0)\right]. \tag{S7}$$

To quantify departure from additive effects from predictions on a relative risk scale, we can compute the relative excess risk due to interaction (RERI). In our context, we can define exposures as "high" vs "baseline" (e.g. t_1 vs t_0, and a_1 vs a_0) and use the odds ratios as approximations for the relative risk predictions. The RERI is then computed using already estimated values with

$$\widehat{RERI} = \widehat{OR}_{11} - \widehat{OR}_{10} - \widehat{OR}_{01} + 1. \tag{S8}$$

2 Exposure distributions

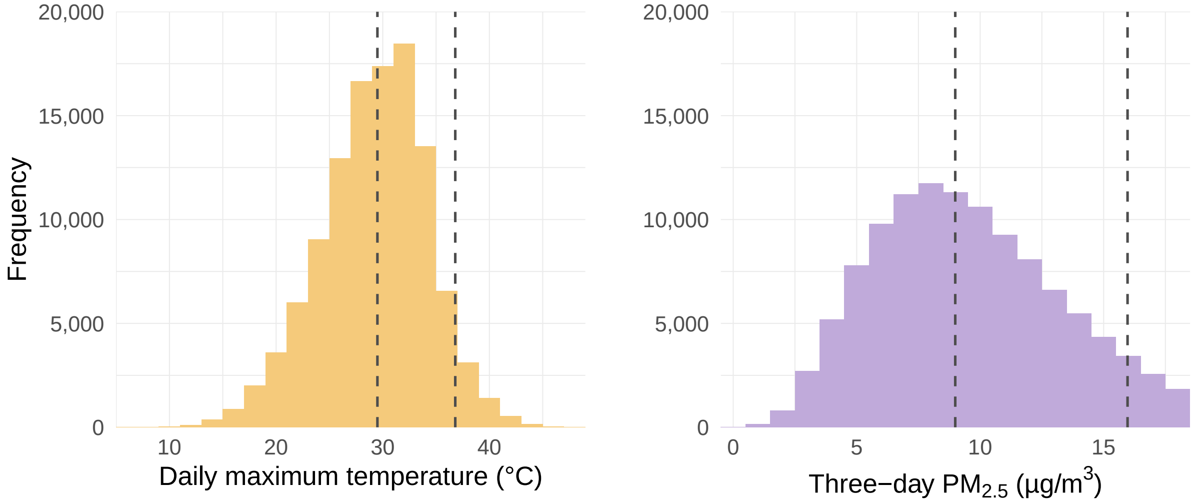

Figure S1: Case day distributions of daily maximum temperature and three-day average $PM_{2.5}$ exposures. The dashed lines indicate the median and 95th percentiles of each exposure, which we use to estimate the RERI. Note that days on which $PM_{2.5}$ exceeded 18.4 $\mu g/m^3$ were excluded from the analysis. Prior to trimming, the maximum case day three-day average $PM_{2.5}$ exposure was 120.4 $\mu g/m^3$.

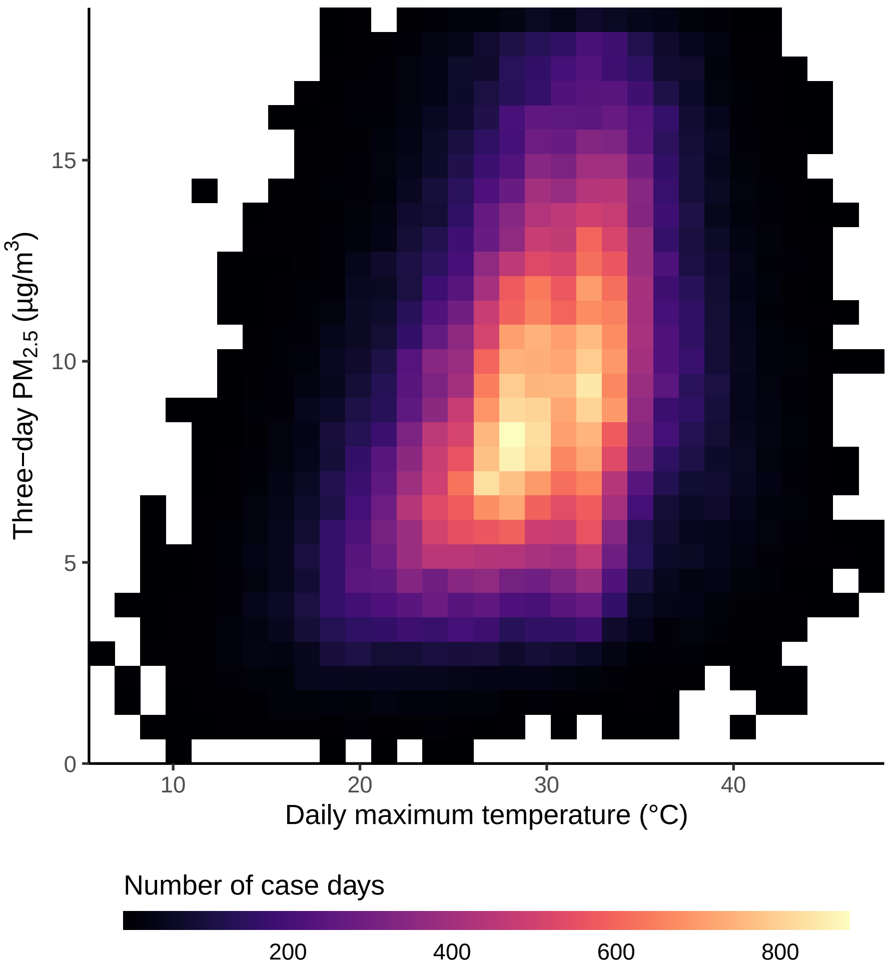

Figure S2: Case day joint daily maximum temperature and three-day average $PM_{2.5}$ exposures. Each case day is assigned to the pixel that covers its temperature and $PM_{2.5}$ exposures; color indicates the number of case days falling within each pixel. White pixels indicate the absence of case days with a given temperature and $PM_{2.5}$ joint exposure.

2.1 Sensitivity analysis results

We conducted three sensitivity analyses. First, we considered three-day average temperature exposures instead of same-day exposures. Second, we trimmed $PM_{2.5}$ exposures at the 99th percentile instead of the 95th percentile. Lastly, we used a tensor product as opposed to natural cubic splines with an interaction term to capture non-linear interaction between exposures.

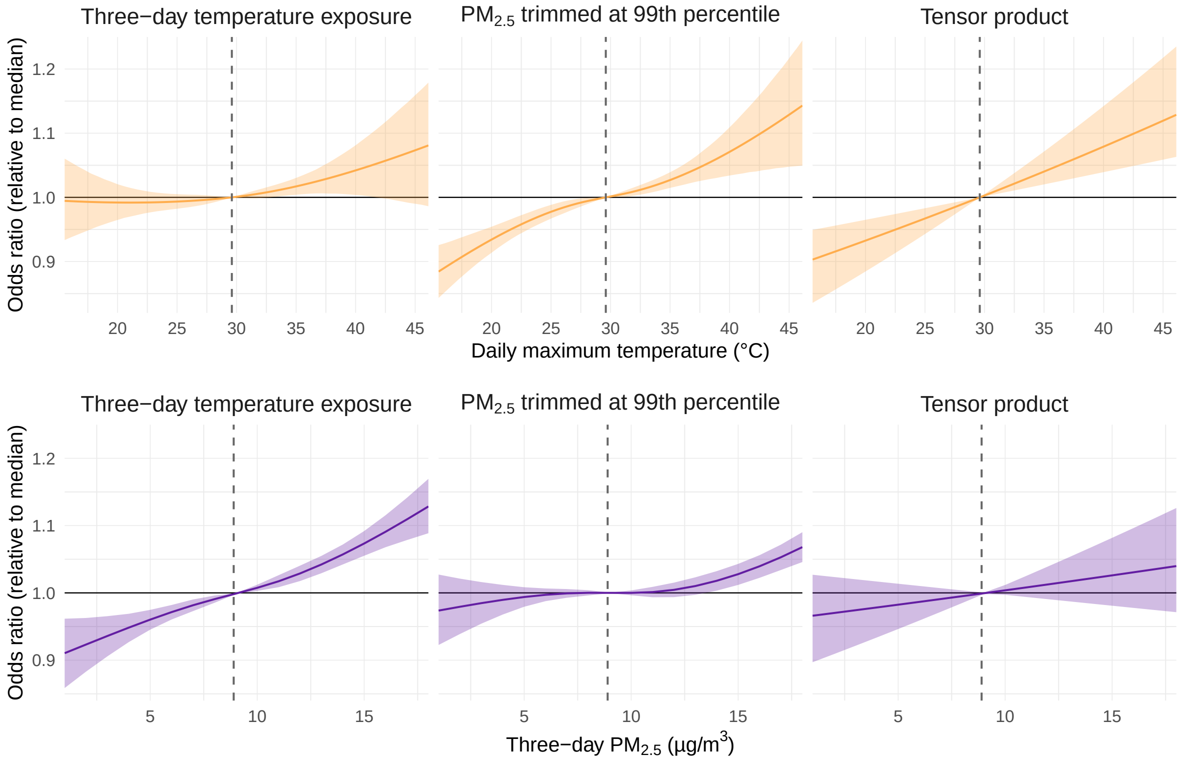

Figure S3: Equivalent to Figure 2 with the independent nonlinear effects of temperature and $PM_{2.5}$ on heat-related hospitalization from the three sensitivity analyses. The top row displays the odds ratio of heat-related hospitalization comparing the median case day temperature (29.6°C), shown with a vertical dashed line, to various alternative temperatures across the x-axis, while holding $PM_{2.5}$ exposure fixed at the median (8.9 $\mu g/m^3$). The bottom row displays the odds ratio of heat-related hospitalization comparing the median case day $PM_{2.5}$ exposure, shown with a vertical dashed line, to various alternative concentration levels across the x-axis, while holding temperature exposure fixed at the median.

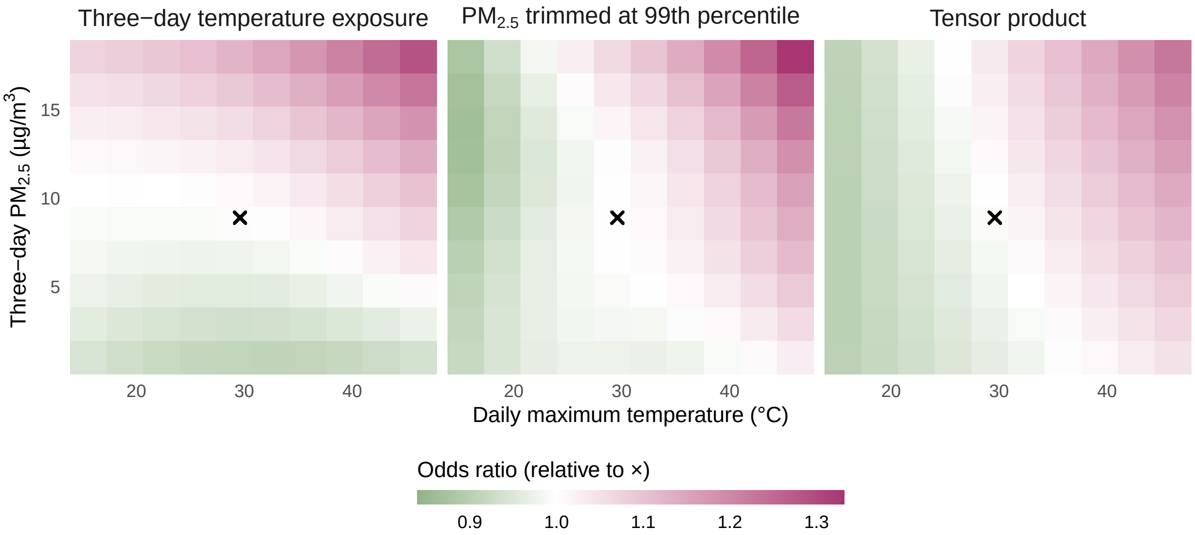

Figure S4: Equivalent to Figure 4 with the synergistic effects of temperature and $PM_{2.5}$ across a range of exposure values from the three sensitivity analyses. The tile color indicates the odds ratio of heat-related hospitalization for a given pair of temperature and $PM_{2.5}$ exposures versus the median temperature and $PM_{2.5}$ exposures from the main analysis (marked on the grid with an ×).

	Estimate (95% credible interval)			
	Main analysis	Three-day temperature	$PM_{2.5}$ trimmed at 99th percentile	Tensor product
OR_{10}	1.05 (1.03, 1.06)	1.03 (1.01, 1.05)	1.04 (1.02, 1.06)	1.05 (1.03, 1.10)
OR_{01}	1.01 (0.99, 1.04)	1.09 (1.07, 1.12)	1.04 (1.02, 1.06)	1.03 (0.98, 1.10)
OR_{11}	1.09 (1.06, 1.12)	1.14 (1.11, 1.18)	1.12 (1.09, 1.14)	1.10 (1.04, 1.19)
RERI	0.03 (0.01, 0.06)	0.02 (-0.00, 0.05)	0.03 (0.01, 0.05)	0.02 (-0.03, 0.07)

Table S2: Odds ratios (OR) and relative excess risk due to interaction (RERI) results from the main analysis and three sensitivity analyses, as presented in Figure 3. Uncertainty intervals are 95% Bayesian credible intervals. OR_{10} indicates the independent effect of temperature, OR_{01} indicates the independent effect of $PM_{2.5}$, and OR_{11} indicates the combined effect of temperature and $PM_{2.5}$. All quantities displayed here are defined above in section 1.4.

References

Pedersen, E. J., Miller, D. L., Simpson, G. L., and Ross, N. (2019). Hierarchical generalized additive models in ecology: an introduction with mgcv. *PeerJ*, 7:e6876.

Wood, S. N., Scheipl, F., and Faraway, J. J. (2013). Straightforward intermediate rank tensor product smoothing in mixed models. *Stat Comput*, 23:341–360.
